# A ‘One Health’ perspective of Africa-wide distribution and prevalence of Giardia species in humans, animals and waterbodies: a systematic review and meta-analysis

**DOI:** 10.1017/S0031182023000513

**Published:** 2023-08

**Authors:** Mpho Tawana, ThankGod E. Onyiche, Tsepo Ramatla, Oriel Thekisoe

**Affiliations:** 1 Unit for Environmental Sciences and Management, North-West University, Potchefstroom 2531, South Africa; 2 Department of Veterinary Parasitology and Entomology, University of Maiduguri, Maiduguri, Nigeria

**Keywords:** animals and waterbodies, *Giardia duodenalis*, humans

## Abstract

Giardiasis, caused by *Giardia duodenalis*, is a leading cause of diarrhoea in resource-poor countries. To gain a better insight into the epidemiology of *Giardia* in Africa, we undertook a robust study to comprehend the distribution and prevalence of *Giardia* infection in humans, animals and their dispersal in the environment. Our protocol was registered with PROSPERO (registration number CRD42022317653). Deep literature search from 5 electronic databases, namely, AJOL, Google scholar, PubMed, ScienceDirect and Springer Link was performed using relevant keywords. Meta-analysis was performed using a random-effects model and heterogeneity among studies was evaluated using Cochran's Q and the *I^2^*-statistic. More than 500 eligible studies published from 1 January 1980 until 22 March 2022 were retrieved. In humans, exactly 48 124 *Giardia* spp. infection cases were registered from the 494 014 stool samples examined resulting in a pooled prevalence estimate (PPE) of 8.8% using microscopy. Whereas copro-antigen tests and molecular diagnostic methods generated PPE of 14.3 and 19.5%, respectively, with HIV+ subjects and those with diarrhoeatic stool having infection rates of 5.0 and 12.3%, respectively. The PPE of *Giardia* spp. infection in animals using molecular methods was 15.6%, which was most prevalent in pigs (25.2%) with Nigeria registering the highest prevalence at 20.1%. The PPE of *Giardia* spp. contamination from waterbodies was 11.9% from a total of 7950 samples which were detected using microscopy, with Tunisia documenting the highest infection rate of 37.3%. This meta-analysis highlights the necessity of ‘One Health’ approach for consolidated epidemiological studies and control of giardiasis in the African continent.

## Graphical abstract

**Figure:**
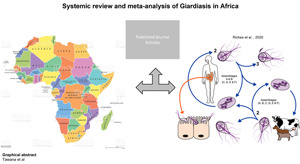

## Introduction

*Giardia* (Metamonada, Giardiidae) is an enteric flagellated diplomonad protozoan parasite that infects a wide range of mammalian hosts including humans and animals, leading to one of the most frequently occurring parasitic diseases known as giardiasis (Xu *et al*., 2020). The infection can be asymptomatic in some cases, and when symptoms do appear, they can range from persistent diarrhoea, abdominal pain, by severe malabsorption, all of which can have a negative influence on growth and intellectual development (Ramírez *et al*., 2015). This protist is ubiquitously distributed and is responsible for cases of human diarrhoea annually, mostly in children <5 years of age with lower prevalence in developed compared to developing countries (Feng and Xiao, 2011; Mahdavi *et al*., 2022) and can infect over 40 animal species (Thompson and Monis, 2004; Taghipour *et al*., 2022). Life cycle of *G. duodenalis* begins when the infective cyst forms are shed into the environment in fecal material; excystation occurs which is enhanced by the gastric acid and pancreatic enzymes after ingestion by another host forming 2 motile pear-shaped trophozoites that subsequently colonize the small intestine (duodenum and jejunum) provoking conjugation and lipid metabolism dysfunction (Li *et al*., 2017; Buret *et al*., 2020).

Ingestion of cysts from polluted water or food causes infection in humans and other mammals (House *et al*., 2011). Transmission of this parasite is direct *via* the fecal–oral route, as in the case of farmers, veterinarians and petting zoos, or indirectly, as in polluted surface water or foods (Hunter and Thompson, 2005; Dixon *et al*., 2011). Water sources infected with cysts from fecal deposition or sewage disposal techniques are the most common sources of *Giardia* infection in humans (Solarczyk *et al*., 2021). Dogs and cats that are kept as pets could also serve as major zoonotic transmission route to humans (Aw *et al*., 2019).

Globally, the detection, identification and characterization of *Giardia* are central to investigating and understanding the epidemiology of giardiasis. Diagnostic methods that have been employed broadly include fecal microscopy, immunodiagnostics and molecular techniques (Hooshyar *et al*., 2019).

Giardiasis has been included in the Neglected Disease Initiatives of the World Health Organization (WHO) since September 2004 due to its health effects on children and pregnant women as well as being associated with poverty (Mirrezaie *et al*., 2019). The incidence of *Giardia* in the resource-poor countries is estimated to be 20–30% due to hazardous water supplies, sanitation and hygiene (Groudan *et al*., 2021). In sub-Saharan Africa and West Africa, prevalences are estimated to be 7.36 and 8.97%, respectively (Bogoch *et al*., 2006; Belhassen-García *et al*., 2017). We performed a systematic review and meta-analysis using a ‘One Health’ approach in order to better define the prevalence and epidemiological distribution of *Giardia* species in animals, humans and waterbodies from published literature between 1980 and 2022 in the African continent. We are of the opinion that the outcomes of this study will be useful to policy makers on how to minimize the burden of giardiasis in developing countries.

## Materials and methods

### Protocol and registration

The protocol for this study was preregistered in PROSPERO with registration number CRD42022317653. We performed a systematic review and meta-analysis following the Preferred Reporting Items for Systematic Reviews and Meta Analyses (PRISMA) guidelines (Page *et al*., 2021) which have been confirmed on a checklist (Supplementary Table S1).

### Search strategy

Literature searches were conducted using keywords (Table 1) on PubMed, ScienceDirect, AJOL, SpringerLink and Google Scholar on articles published in English language from 1 January 1980 until 22 March 2022 for articles with emphasis on the prevalence or epidemiology of *Giardia* species across the continent of Africa in animals, humans and waterbodies. None of the authors of original studies were contacted for additional information and no attempt was made to retrieve unpublished articles. Titles and abstracts were scanned, and relevant full-text articles were downloaded and obtained through library resources and online platforms.

**Table 1.** Search strategy

Databases	Search strategy
Google scholar	‘Intestinal Parasites’ OR ‘Opportunistic pro-tozoa’ OR ‘*Giardia*’ OR ‘Giardiasis’ AND ‘Prevalence’ OR ‘Epidemiology’ OR ‘Frequency’ AND ‘Human’ OR ‘Animal’ OR ‘Waterbodies’
PubMed	‘Intestinal Parasites’ OR ‘Enteropathogen’ OR ‘Opportunistic protozoa’ OR ‘*Giardia*’ OR ‘Giardiasis’ AND ‘Prevalence’ OR ‘Epidemiology’ OR ‘Frequency’ AND ‘Human’ OR ‘Animal’ OR ‘Waterbodies’ in Africa
AJOL	‘Intestinal Parasites’ OR ‘Enteropathogen’ OR ‘Opportunistic protozoa’ OR ‘*Giardia*’ OR ‘Giardiasis’ AND ‘Prevalence’ OR ‘Epidemiology’ OR ‘Frequency’ AND ‘Human’ OR ‘Animal’ OR ‘Waterbodies’ in Africa
ScienceDirect	‘Intestinal Parasites’ OR ‘Enteropathogen’ OR ‘Opportunistic protozoa’ OR ‘*Giardia*’ OR ‘Giardiasis’ AND ‘Prevalence’ OR ‘Epidemiology’ OR ‘Frequency’ AND ‘Human’ OR ‘Animal’ OR ‘Waterbodies’ in Africa
Springer Link	‘Intestinal Parasites’ OR ‘Enteropathogen’ OR ‘Opportunistic protozoa’ OR ‘*Giardia*’ OR ‘Giardiasis’ AND ‘Prevalence’ OR ‘Epidemiology’ OR ‘Frequency’ AND ‘Human’ OR ‘Animal’ OR ‘Waterbodies’ in Africa

### Eligibility criteria

#### Inclusion and exclusion criteria

Articles were included only if they fulfilled the following inclusion criteria: cross-sectional (prevalence) study conducted within African continent; study involving the detection and/or screening of vertebrate hosts (humans or animals), waterbodies including fecal and water samples for *Giardia duodenalis* (sny. *G. intestinalis*, *G. lamblia*); the exact total numbers and positive cases were clearly provided; sample size (>50 to enable statistical computations); published study written in English language; study conducted between 1 January 1980 until 22 March 2022. Studies were excluded if (i) they were conducted outside of Africa, (ii) case control or randomized studies, (iii) involved the detection of *Giardia* in fresh produce or soil, (iv) incomplete data on the total number of samples screened or number of positives obtained, (v) published papers outside the study periods and (vi) written in other languages.

### Study selection and data extraction

Independent reviewers (M. T. and T. O.) carefully evaluated all titles and abstracts identified in the search, as well as full texts considered to be relevant. Any disagreements were resolved by discussion with the other 2 authors (T. R. and O. T.). Titles and abstracts derived through primary electronic search were thoroughly assessed for possibility of inclusion based on the study type (prevalence of *Giardia* in animals, humans and waterbodies), and duplicates were removed. Full texts were examined and unrelated studies were excluded with reasons. All studies that met the eligibility criteria were included for syntheses. From each eligible study, the following data were extracted and organized using Microsoft Excel spreadsheet using the format: name of the author and countries, study/publication year, country, hosts, total sample size, number of positive cases, estimated prevalence, consistency of the feces and different diagnostic technique. Studies that were conducted in more than 1 country and those that had both animal, human and waterbodies studies simultaneously were separated accordingly.

### Quality assessment of included studies/risk of bias

The risk of bias for each study was assessed using the Joanna Briggs Institute (JBI) Critical Appraisal Tools for cross-sectional study (Munn *et al*., 2015). This JBI instrument consists of 9 criteria, of which details are available (Supplementary Table S2). Each response to the individual criteria was assigned a score of 0 or 1 for no or yes answers. When the question was not applicable to the study, not applicable (NA) was used. A maximum score of 9 was possible but only 8 was applicable to our kind of study that was eligible for incorporation in this review. Studies with scores of 7–8 indicated a low risk of bias, scores of 5–6 indicated a moderate risk of bias, and scores less than 5 indicated a high risk of bias.

### Data synthesis

The current meta-analysis was conducted using the Comprehensive Meta-Analysis software (CMA) version 3.0 software (Borenstein *et al*., 2014). The pooled prevalence estimates (PPE) and 95% confidence interval (CI) were calculated using random-effects models. Statistical heterogeneity between studies was measured by *I*^2^ statistic (Higgins *et al*., 2003). Publication bias was measured using funnel plots to test for symmetry and this was further complimented using the Begg's and Mazumdar rank (BMR) correlation test (Begg and Mazumdar, 1994).

## Results

### Search results

**Figure 1.**
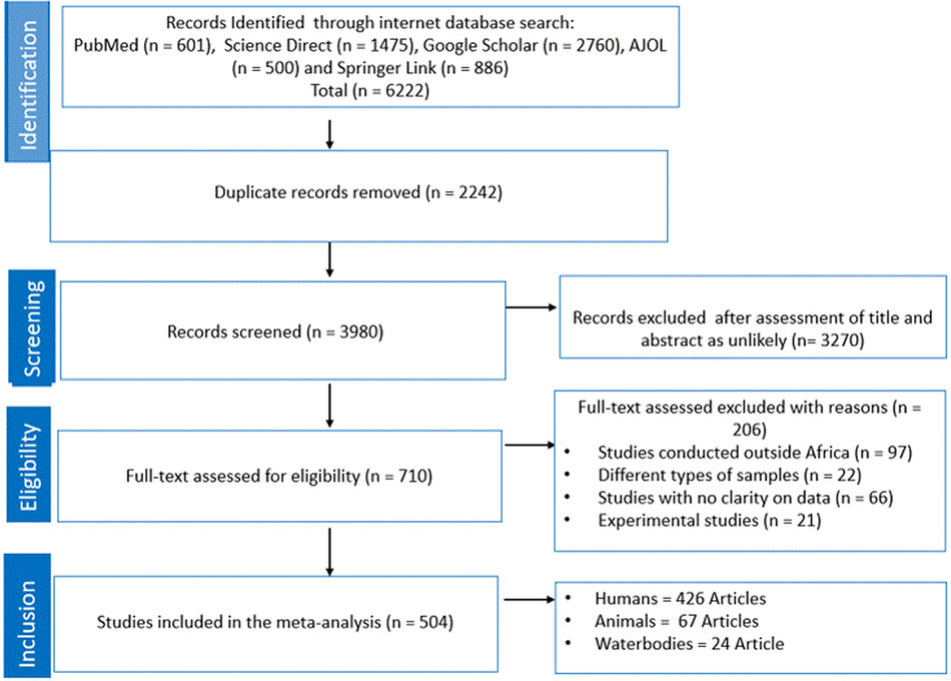
PRISMA flow chart.

A total of 6222 studies were retrieved following the initial search from 5 databases (Fig. 1). A total of 2242 articles were removed as they were duplicates and the remaining articles (*n* = 3980) were screened based on titles and abstracts. Thereafter, a total of 3270 was removed as unlikely leaving 710 studies that were subjected for eligibility and were thus examined by full-text evaluation. Exactly 206 articles were excluded with reasons as follows: studies conducted outside of Africa (*n* = 97), studies with no clarity on data (*n* = 66), studies with focus on animal experiment (*n* = 21) and lastly, studies with different types of samples (*n* = 22). Ultimately, for the quantitative synthesis (meta-analysis) of eligible studies, a total of 426, 67 and 24 studies were used for human, animal and waterbodies, respectively, to obtain the PPE (Fig. 1).

### General characteristics of the included studies

The characteristics of all eligible studies included in this review are presented in Supplementary Tables S3–S5. All studies were published from 1980 to 2022, with the majority of the studies conducted in the last 2 decades (2002–2022). The prevalence for all the individual studies was computed and presented in Supplementary Table S1. All studies were conducted in different parts of Africa, with eligible studies with focus on humans widely distributed across the continent in different countries. Exactly 34 countries documented the existence of *Giardia* species in samples collected from humans with Ethiopia (*n* = 115) having the highest number of studies, followed by Nigeria and Egypt with 53 studies each. Others include Kenya (*n* = 31), Ghana (*n* = 26), Guinea (*n* = 15) and South Africa (*n* = 14). Details of the findings from other countries are presented in Fig. 1. For studies pertaining to animals, Uganda (*n* = 11) and Nigeria (*n* = 9) had high number of eligible studies. Lastly, both Egypt and South Africa had 6 published eligible studies with interest on *Giardia* species from samples collected from waterbodies. Diagnostic technique employed across the 3 (human, animals and waterbodies) different subjects of interest includes microscopy, copro-antigen tests and molecular-based diagnostics.

### Quality assessment of included studies

The quality assessment score of included studies ranged from 7 to 8 as per JBL critical appraisal checklist for studies reporting *Giardia* spp. prevalence data. About 10, 6 and 11 studies were excluded from this systematic review and meta-analysis for humans, animals and waterbodies, respectively, since they scored less than 66.7%.

### PPE of *G. duodenalis* infection in humans

Different diagnostic methods were utilized for the detection of *Giardia* spp. infections from stool samples collected from humans across the continent. Of the 494 014 stool samples examined for *Giardia* spp. infection, 48 124 cases were registered as positives using microscopy. Thus, the PPE was 8.8% (95% CI 8.0–9.6%) (Table 2). Using copro-antigen tests, the PPE of anti-*Giardia* spp. was 14.3% (95% CI 10.1–20.0%). Lastly, a total of 16 095 stool samples were examined for the prevalence of *Giardia* spp. infection using molecular-based methods, out of which 2437 samples were positive with PPE of 19.5% (95% CI 13.0–24.3%) (Table 2). According to gender, the PPE in males was 13.3% (95% CI 11.7–15.1%) compared to 11.9% (95% CI 10.3–13.8%) in females. Furthermore, *G. duodenalis* infections were more prevalent in human subjects within the age range 0–18 years with PPE of 13.4% (95% CI 10.9–16.4%) while those within 19–35 years had the least PPE of 8.8% (95% CI 7.1–10.8%). The PPE in human subjects in rural areas was 17.9% (95% CI 13.0–24.3%) comparatively higher to human subjects in urban areas 9.2% (95% CI 6.3–13.1%) (Table 2). Additionally, the PPE of giardiasis was notably observed in the 1981–1990 year interval at 11.4% (95% CI 7.5–17.0%), followed by 9.6% (95% CI 7.4–12.3%) in 2001–2010, 9.6% (95% CI 8.6–10.6%) in 2011–2022 and 7.9% (95% CI 5.4–11.6%) in 1990−2000 year interval (Table 2). Human subjects that were HIV+ had a higher PPE of giardiasis at 5.0% (95% CI 3.5–7.3%) compared to those who were HIV– at 4.1% (95% CI 2.5–6.6%). Based on stool consistency, patients with diarrhoetic stool had a higher PPE (12.3%; 10.3–14.7%) compared to non-diarrhoetic patients (9.7%; 7.2–12.9%). Finally, the PPEs at country level indicate that Tunisia registered the highest at 39.9% (with only 2 eligible studies) while the lowest PPE was registered in Cameroon 1.3% (95% CI 0.4–4.1%) (Table 2).

**Table 2.** Sub-group analysis of *Giardia duodenalis* infection in human subjects across Africa

Subgroup variable	Number of studies	Pooled prevalence estimates			Measure of heterogeneity			Publication bias
		Sample size	Number of positive	Prevalence 95% CI (%)	*Q*	*I*^2^	*Q-P*	Begg and Mazumdar rank (*P* value)
Diagnostic method								
Microscopy	401	494 014	48 124	8.8 (8.0–9.6)	31 514.51	98.73	0.000	0.000
Serology	9	6145	1424	14.3 (10.1–20.0)	136.64	94.15	0.000	0.425
Molecular technique	17	16 095	2437	19.5 (10.8–32.6)	2531.14	99.37	0.000	0.282
Sex								
Female	89	54 160	7071	11.9 (10.3–13.8)	2852.01	96.91	0.000	0.004
Male	88	54 520	6987	13.3 (11.7–15.1)	2203.34	96.01	0.000	0.008
Age (years)								
0–18	58	58 365	6615	13.4 (10.9–16.4)	3494.93	98.37	0.000	0.040
19–35	28	21 429	2554	8.8 (7.1–1 0.8)	416.00	93.75	0.000	0.095
> 36	20	11 465	1287	9.4 (6.5–13.5)	338.40	94.39	0.000	0.030
Human setting								
Rural region	17	6769	1183	17.9 (13.0–24.3)	495.28	96.77	0.000	0.467
Urban region	16	4118	426	9.2 (6.3–13.1)	206.59	92.26	0.000	0.108
Study year								
1981–1990	20	27 185	3176	11.4 (7.5–17.0)	2268.01	99.16	0.000	0.248
1991–2000	27	27 476	2649	7.9 (5.4–11.6)	2158.18	98.80	0.000	0.117
2001–2010	82	197 661	11 390	9.6 (7.4–12.3)	9705.30	99.17	0.000	0.061
2011–2022	197	202 130	26 492	9.6 (8.6–10.6)	11 666.71	98.32	0.000	0.000
HIV status								
HIV+	51	21 710	7078	5.0 (3.5–7.3)	1591.16	96.67	0.000	0.004
HIV−	18	5001	210	4.1 (2.5–6.6)	138.87	87.76	0.000	0.007
Stool consistency								
Diarrhoeic	82	76 656	11 600	12.3 (10.3–14.7)	5322.73	98.48	0.000	0.001
Non-diarrhoeic	59	23 863	3371	9.7 (7.2–12.9)	4433.19	98.69	0.000	0.137
Country								
Algeria	5	9733	1504	16.7 (9.1–28.7)	364.02	98.90	0.000	0.500
Angola	5	2307	406	19.3 (10.5–32.9)	162.20	97.53	0.000	0.071
Botswana	5	5618	152	4.0 (1.1–13.3)	195.33	97.95	0.000	0.312
Burkina Faso	7	13 904	5364	14.2 (6.5–28.5)	597.91	99.00	0.000	0.088
Cameroon	5	1472	22	1.3 (0.4–4.1)	22.54	82.26	0.000	0.014
Chad	2	1081	257	23.8	-	-	-	-
Cote d'Ivoire	13	10 721	1641	18.2 (15.7–20.9)	106.96	88.78	0.000	0.232
DR Congo	3	1212	64	4.8 (2.5–9.0)	11.28	82.27	0.000	0.301
Egypt	53	30 220	5878	16.9 (13.9–20.5)	2772.16	98.12	0.000	0.255
Ethiopia	115	158 683	17 500	7.4 (6.6–8.1)	3611.11	96.84	0.000	0.000
Gabon	7	1434	147	11.3 (6.9–17.9)	50.81	88.19	0.000	0.274
Gambia	3	279	45	15.3 (5.4–36.6)	20.59	90.28	0.000	0.059
Ghana	26	10 149	1836	6.4 (4.0–10.2)	1521.33	98.36	0.000	0.430
Guinea	15	13 485	2769	19.2 (11.9–29.4)	1790.16	99.22	0.000	0.402
Kenya	31	24 427	2701	8.8 (5.9–13.1)	2736.95	98.90	0.000	0.373
Lesotho	4	667	98	12.9 (5.9–26.0)	33.42	91.02	0.000	0.087
Libya	5	4611	329	6.2 (2.7–13.6)	124.99	96.80	0.000	0.312
Malawi	3	954	280	29.4 (26.6–32.3)	0.58	0.00	0.000	0.30
Madagascar	2	2957	335	11.3				
Morocco	3	2349	181	8.2 (4.5–14.5)	32.46	93.84	0.000	0.301
Mozambique	11	11 075	3754	20.4 (12.3–31.8)	1542.12	99.35	0.000	0.469
Nigeria	53	170 133	2002	4.6 (3.0–6.9)	3591.91	98.55	0.000	0.000
Rwanda	8	4090	1468	26.2 (15.6–40.5)	540.79	98.71	0.000	0.161
Senegal	6	4665	337	8.9 (2.7–25.4)	496.91	98.99	0.000	0.094
Sahrawi	2	390	119	30.5				
Sao Tome	4	2469	505	21.5 (9.9–40.8)	233.08	98.71	0.006	0.087
Sierra Leone	2	647	170	26.3				
South Africa	14	13 824	1319	9.5 (6.2–14.3)	618.57	97.90	0.000	0.392
Sudan	7	2378	268	10.7 (6.2–17.9)	118.00	94.92	0.000	0.025
Tanzania	12	8075	722	7.6 (4.4–12.9)	490.04	97.76	0.000	0.340
Tunisia	2	414	165	39.9				
Uganda	12	3943	379	9.4 (5.7–15.3)	237.64	95.37	0.000	0.037
Zambia	6	6466	457	9.5 (2.8–27.6)	592.78	99.16	0.001	0.174
Zimbabwe	3	2753	804	29.5 (9.1–63.7)	407.42	99.51	0.235	0.301

### PPE of *G. duodenalis* infection in animals

Just like in human subjects, 3 diagnostic techniques were utilized in the detection of *Giardia* spp. infection in humans. Of the 12 873 fecal samples screened from animals using microscopy, a total of 856 were positive with PPE at 5.8% (95% CI 4.4–7.6%) (Table 3). Using serology, 789 samples were positive to anti-*Giardia* spp. out of a total of 3896 samples screened with an estimated PP at 17.7% (95% CI 10.7–27.7%) (Table 3). From a total of 5515 samples, 922 were positive for *Giardia* spp. infections using molecular methods with a PPE of 15.6% (95% CI 9.4–24.9%) (Table 3). According to study year, *Giardia* spp. infections were more prevalent in the 2011–2022 year interval with PPE of 16.2% (95% CI 11.0–23.3%), followed by 8.9% (95% CI 5.7–13.6) in 2001–2010, and then 2.6% (95% CI 1.0–6.9%) in 1990–2000 year interval (Table 3). Based on animal host, pigs had the highest PPE of 25.2% (95% CI 9.7–51.6%) followed by goats of 18.9% (95% CI 11.5–29.4) and the lowest was observed in monkeys of 5.3% (95% CI 0.8–28.0%) (Table 3). The PPE according to country level indicates that Nigeria had the highest at 20.1% (95% CI 10.8–34.1%) and Rwanda with the lowest at 4.3% (95% CI 2.9–6.3%) (Table 3).

**Table 3.** Sub-group analysis of *Giardia* species infection in animals' species across Africa

Subgroup variable	Number of studies	Pooled prevalence estimates			Measure of heterogeneity		*Q-P*	Publication bias
		Sample size	Number of positive	Prevalence 95% CI (%)	*Q*	*I*^2^		Begg and Mazumdar rank *P* value
Diagnostic methods								
Microscopy	37	12 873	856	5.8 (4.4–7.6)	495.14	92.72	0.000	0.065
Serology	14	3896	789	17.7 (10.7–27.7)	501.95	97.41	0.000	0.109
Molecular technique	19	5515	922	15.6 (9.4–24.9)	676.02	97.49	0.000	
Study year								
1990–2000	4	2201	39	2.6 (1.0–6.9)	26.61	88.73	0.000	0.248
2001–2010	19	6478	578	8.9 (5.7–13.6)	470.80	96.96	0.000	0.040
2011–2022	25	8069	1579	16.2 (11.0–23.3)	1158.92	97.93	0.000	0.329
Animal species								
Cattle	23	8587	757	8.2 (5.6–11.8)	521.99	95.79	0.000	0.009
Chimpanzee	4	791	86	6.5 (2.1–18.6)	37.58	92.02	0.000	0.249
Dog	4	1047	111	7.4 (2.4–20.4)	70.11	95.72	0.000	0.249
Domestic animals	36	11 546	1228	10.7 (8.1–14.0)	1105.65	96.29	0.000	0.068
Goats	7	995	214	18.9 (11.5–29.4)	71.96	91.66	0.000	0.226
Gorillas	9	976	57	5.4 (2.7–10.5)	46.58	82.82	0.000	0.059
Monkeys	3	2894	199	5.3 (0.8–28.0)	110.69	98.19	0.004	0.301
Pigs	5	1198	222	25.2 (9.7–51.6)	175.30	97.72	0.064	0.312
Sheep	8	1759	195	9.7 (4.8–18.8)	145.35	95.18	0.000	0.402
Wildlife	29	6671	606	8.8 (5.7–13.4)	626.75	95.37	0.000	0.058
Country								
Algeria	3	1119	129	13.4 (6.7–24.9)	25.85	92.26	0.000	0.301
Egypt	7	2838	525	7.2 (1.8–24.6)	544.89	98.90	0.000	0.440
Ethiopia	6	2618	346	7.4 (2.9–17.6)	237.83	97.90	0.000	0.019
Ghana	4	1452	130	7.9 (5.0–12.3)	13.50	77.78	0.000	0.249
Nigeria	9	1828	344	20.1 (10.8–34.1)	232.85	96.56	0.000	0.417
Rwanda	3	598	25	4.3 (2.9–6.3)	1.16	0.00	0.000	0.301
Tanzania	5	1713	101	5.3 (1.6–16.2)	79.79	94.99	0.000	0.164
Uganda	11	3077	191	7.0 (2.6–17.5)	340.03	97.06	0.000	0.349

### PPE of *G. duodenalis* contamination in waterbodies

A total of 7950 samples from various waterbodies across the continent were examined for the prevalence of *Giardia* spp. contamination, out of which 1407 water samples were positive using microscopy with PPE of 11.9% (95% CI 7.7–18.0%) (Table 4). Furthermore, of the 1288 water samples examined for the prevalence of *Giardia* spp. contamination, a total of 454 samples were positive using molecular methods, with PPE of 32.0% (95% CI 24.7–40.2%). The *Giardia* parasite was most prevalent in the 2011–2022 year interval, with PPE of 25.3% (95% CI 13.8–41.6%) as compared to 2001–2010 year interval with 8.9% (95% CI 2.6–26.8%). Finally, based on country distribution, Tunisia registered the highest PPE at 37.3% while the lowest was observed in Nigeria at 15.4% (95% CI 6.2–33.5%) (Table 4).

**Table 4.** Subgroup analysis of *Giardia* species contamination in waterbodies across Africa

Subgroup variables	Number of studies	Pooled prevalence estimates			Measure of heterogeneity		*Q-P*	Publication bias
		Sample size	Number of positive	Prevalence 95% CI (%)	*Q*	*I*^2^		Begg and Mazumdar rank *P* value
Diagnostic methods								
Microscopy	17	7950	1407	11.9 (7.7–18.0)	786.03	97.96	0.000	0.253
Molecular method	6	1288	454	32.0 (24.7–40.2)	24.86	79.89	0.000	0.174
Study year								
2001–2010	5	1150	75	8.9 (2.6–26.8)	105.74	96.22	0.000	0.071
2011–2022	6	2911	530	25.3 (13.8–41.6)	168.46	97.03	0.004	0.174
Countries								
Egypt	6	2218	119	8.0 (1.5–33.6)	182.63	97.26	0.007	0.287
Ethiopia	2	111	64	57.7	–	–	–	–
Nigeria	3	987	186	15.4 (6.2–33.5)	21.32	90.62	0.001	0.301
South Africa	6	2327	797	28.4 (18.5–40.8)	138.52	96.39	0.001	0.174
Tunisia	2	271	101	37.3	–	–	–	–
Uganda	2	871	296	33.9	–	–	–	–

### Risk of publication bias of included studies

**Figure 2.**
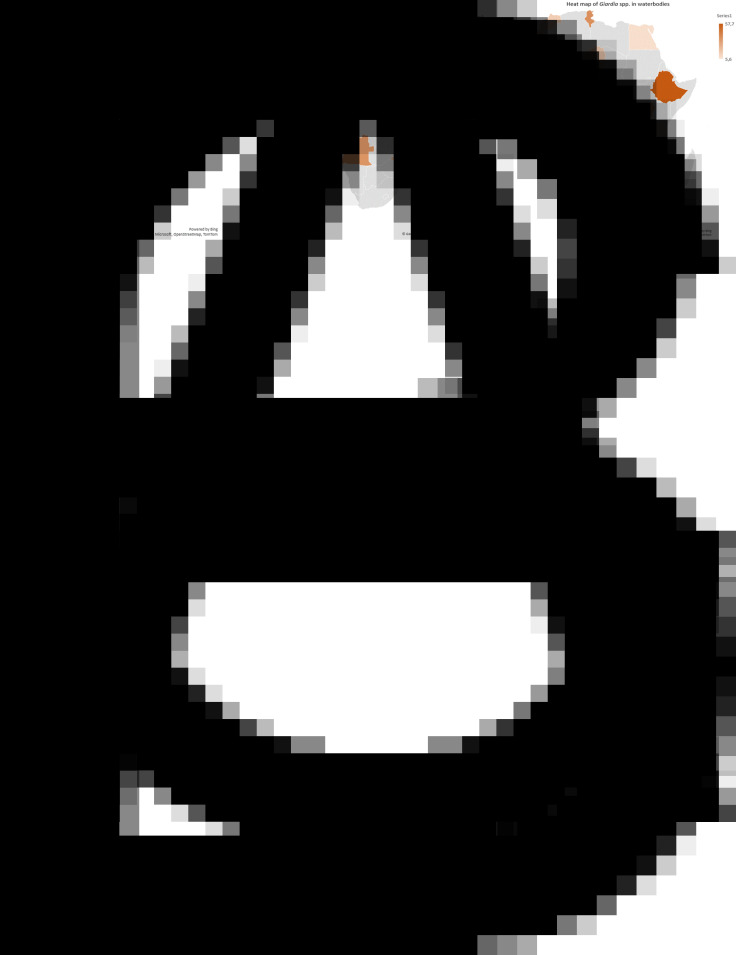
Heat maps showing pooled prevalence estimates of *Giardia* spp. per country (A) humans, (B) animals and (C) waterbodies.

The funnel plots of the estimates suggested publication bias (Supplementary Figs S1–S8) for both human studies with asymmetric presentations and BMR test values observed with respect to human studies were microscopy method (*P* = 0.000), males (*P* = 0.004), females (*P* = 0.008), 2011–2022 year interval (*P* = 0.000), HIV+ (*P* = 0.004), HIV– (*P* = 0.007), diarrhoeic (*P* = 0.001), Cameroon (*P* = 0.014), Ethiopia (*P* = 0.000), Nigeria (*P* = 0.000) and Sudan (*P* = 0.025). Whereas for animals, observed publication bias was on 2001–2010 year interval (*P* = 0.040), cattle (*P* = 0.009) and Ethiopia (*P* = 0.019) (Tables 3 and 4 and Supplementary Figs S9–S11). However, Funnel plots and BMR tests suggested no publication bias for waterbody studies (Fig. 2).

## Discussion

Herein, we carried out a robust study to investigate the epidemiology and occurrence of *Giardia* species in humans, animals and from waterbodies across Africa using over 500 published articles that employ different diagnostic methods including microscopy, copro-antigen and molecular-based techniques. Arising from differences in the sensitivity and specificity of the diagnostic methods employed in the investigation of the prevalence of giardiasis, we observed varying prevalence rates (Hooshyar *et al*., 2019). Light microscopy is regarded as the gold standard method recommended for the diagnosis of cystic and/or trophozoite stages of *G. duodenalis* (Soares and Tasca, 2016). Several factors including number of fecal samples and operator experience examined may affect the outcome of investigation of giardiasis using this approach as opined by Elmi *et al*. (2017) and David and AP (2006) leading to lower prevalence of infection or complete failure of detection (Taghipour *et al*., 2022). There is an increased utilization of immunodiagnostic techniques to complement fecal microscopy for giardiasis detection and appears to be reliable for confirmatory diagnosis (Hooshyar *et al*., 2019). On the other hand, DNA-based molecular methods are also reported to be more reliable with appropriate sensitivity for the identification of *Giardia* species (Thompson and Monis, 2004; Gelanew *et al*., 2007).

### Giardia species infection in humans

The total PPE of *Giardia* parasite infections in humans was higher using molecular techniques (19.5%) as compared to microscopic (8.8%) and copro-antigen (14.3%), a finding that is similar to that documented in Ghana and Spain, respectively, where molecular tests (6.6 and 37.5%) detected more *Giardia* spp.-positive infections than microscopy (1.0 and 4.1%) and copro-antigen (5.6 and 4.1%) (Anim-Baidoo *et al*., 2016; Alharbi *et al*., 2020). However, Al-Shehri *et al*. (2016) in Uganda (41.6; 19.3; 0%), Gasparinho *et al*. (2017) in Angola (18.8; 4.1; 0.5%) and Jelinek and Neifer (2013) in Germany (51.1; 50.0; 46.6%) reported that serological tests detected more positives as compared to microscopic and molecular techniques in that order. Whereas in Gambia, Goudal *et al*. (2019) and Sullivan *et al*. (1991) reported that microscopy (20.1 and 48.6%) detected more *Giardia* spp.-positive infections as compared to serological tests (18.5 and 30.6%) in that order respectively. Furthermore, Emisiko *et al*. (2020) in Kenya (46.5; 13.0%), Geus *et al*. (2019) in Rwanda (36.0; 7.1%) and El Fatni *et al*. (2014) in Morocco (12.5; 3.0%) reported that microscopy detected more *Giardia* spp.-positive infections as compared to molecular methods. Remarkably, Becker *et al*. (2015) reported similar detection prevalence (28.7%) by microscopy, copro-antigen and molecular methods in Côte d'Ivoire. Van den Bossche *et al*. (2015) (4.1; 4.1%) in Belgium and Doni *et al*. (2013) (19.6; 19.6%) in Turkey reported equal prevalence on microscopic and copro-antigen methods and Irisarri-Gutiérrez *et al*. (2017) in Mozambique (6.1; 7.1%) and El-Badry *et al*. (2017) in Egypt (11.0; 11.9%) reported microscopic and copro-antigen methods to have similarly equal results towards molecular methods, respectively. This difference can be accounted by differences in specificity and sensitivity of detection techniques used and our review showed that prevalences generally are higher when molecular detection methods are used compared to microscopic or copro-antigen tests, suggesting that molecular tests have a higher sensitivity.

Country-specific findings indicate that Tunisia had the highest PPE of 39.9% for *Giardia* spp. infections, with the rural populations (17.9%) being more susceptible as compared to the population in urban regions (9.2%). This observation is similar to that reported by Díaz *et al*. (2015) in Peru (53.1% rural; 40.6% urban), El Fatni *et al*. (2014) in Morocco (19.6% rural; 7.6% urban), Cervantes Gracia *et al*. (2017) in Mexico (22.3% rural; 17.9% urban), Heimer *et al*. (2015) in Rwanda (43.9% rural; 8.7% urban) and Lobo *et al*. (2014) in DR Congo (18.7% rural; 1.9% urban). In contrast, Huot *et al*. (2016) and Loewenson *et al*. (1986) reported higher prevalence of *Giardia* parasite infections in peri-urban regions (12.3; 22.3%) as compared to rural regions (7.2; 15.6%) in Cambodia and Zimbabwe in that order respectively. However, Ahmad *et al*. (2020), Berrilli *et al*. (2006), Ngonjo *et al*. (2015) and Roche and Benito (1999) reported similar *Giardia* spp. prevalence between urban (20.8; 44; 6.9; 7.2%) and rural (21.6; 44; 7.4; 8.6%) area populations in Egypt, Albania, Kenya and Guinea-Bissau, respectively. About 40.0% of the population in Africa resides in urban areas and thus, anthropogenic activities might be responsible for this observation. Furthermore, the wide variety of socio-economical, climate and geographical characteristics might have influenced this geographical difference in *Giardia* species prevalence (Ahmed *et al*., 2018). The infection was higher in male (13.3%) subjects compared to females (11.9%). Similarly, Abdel-Aziz *et al*. (2010), Anim-Baidoo *et al*. (2016), Bauhofer *et al*. (2020), Dacal *et al*. (2018) and Díaz *et al*. (2015) indicated that *Giardia* spp. infections were more common in males (37.8; 7.1; 10.5; 43.0; 51.5%) as compared to females (28.0; 3.9; 8.5; 34.2; 35.8%) in Sudan, Ghana, Mozambique, Angola and Peru, respectively. Akinbo *et al*. (2010), Bayoumy *et al*. (2016), Júlio *et al*. (2012) and Kasaei *et al*. (2018) reported similar prevalence of *Giardia* spp. among males (0.0; 3.9; 6.9; 50%) and females (0.1; 3.8; 6.5; 50%) in Portugal and Iran, respectively. However, in Uganda, South Africa, Ethiopia and Turkey, the prevalence of *Giardia* spp. was lower (15.9; 32.5; 13.7;18.2%) for males and (22.7; 67.5; 19.8; 20.8%) for females (Ali *et al*., 1999; Jarmey-Swan *et al*., 2001; Doni *et al*., 2013; Al-Shehri *et al*., 2016). Environmental risk factors such as work and sports activities may potentially be contributing on the higher male prevalence of *Giardia* spp. in the African continent.

Our results also revealed that the *Giardia* spp. infection was predominantly among children <6 months to 18 years with PPE of 13.4%, compared to adults >36 years (PPE 9.4%). Our findings corroborate observations from different researchers from different countries as documented by Belkessa *et al*. (2021) in Algeria (81.8%), Casalino *et al*. (1988) in Somalia (4.1%), El-Mohammady *et al*. (2012) in Egypt (19.1%) and Rafiei *et al*. (2020) in Iran (12.7%) where they reported that children <10 to 20 years were more susceptible than other age groups. On the contrary, Berhe *et al*. (2018) and Esrey *et al*. (1989) in Ethiopia and Lesotho, respectively, registered higher *Giardia* spp. prevalence in adults aged 19–35 years compared to other age groups. The high prevalence in children can be attributed to unhygienic practices, which include not washing their hands, biting their nails and walking barefoot (Molina *et al*., 2011). We observed a 3.5% decline of *Giardia* spp. infections in humans between 1980–1990 and 1991–2000 which was followed by an increase of 1.7% during 1991–2000 and 2001–2010. These periodic fluctuations across the continent suggest possible inconsistencies in the personal hygiene, exposure to infected animals, consumption of contaminated food and water, as well as inadequate surveillance of *Giardia* parasite infections.

Furthermore, this study found that HIV+ individuals (5.0%) were more infected with *Giardia* spp. as compared to HIV– subjects (4.1%). These findings concur with the reports by Babatunde *et al*. (2010), Bailey *et al*. (2006), Feitosa *et al*. (2001), Liu *et al*. (2019) and Oguntibeju (2006) where they recorded higher prevalence of *Giardia* parasite infections in HIV+ (17.7, 5.3; 4.9; 2.8; 16.7%) as compared to HIV– (5.6; 0.8; 2.4; 0; 10.0%) individuals in Nigeria, South Africa, Brazil, Guangxi and Lesotho, respectively. In contrast, in Guinea-Bissau, Honduras and Senegal, the *Giardia* parasite infection prevalence was slightly higher in HIV– (20.0; 12.5; 2.9%) as compared to HIV+ (8.6; 1.9; 1.7%) patients (Lindo *et al*., 1998; Gassama *et al*., 2001; Roka *et al*., 2012). It is well known that HIV-infected individuals have immuno-compromised system due to loss of CD4 T cells, making them more vulnerable to a variety of illnesses (Faria *et al*., 2017) including opportunistic *Giardia* parasite.

The current study revealed that diarrhoeic individuals (12.3%) had higher *Giardia* spp. infection prevalence than non-diarrhoeal individuals (9.7%). Our findings agree with the report from Algeria (14.6 and 0.3%), India (16.0 and 8.0%), Libya (26.3 and 0.0%) and Madagascar (12.6 and 7.7%) where diarrhoeic individuals had higher *Giardia* parasite infection prevalence as compared to non-diarrhoeal individuals (Dwivedi *et al*., 2007). In contrast, Bodhidatta *et al*. (2010), Haque *et al*. (2005), Messa *et al*. (2021) and Tellevik *et al*. (2015) found that *Giardia* spp. prevalence was higher in non-diarrhoeal (10.0; 18.0; 32.0; 6.1%) as compared to diarrhoeal (6.0; 7.7; 20.0; 3.4%) individuals in Thailand, Bangladesh, Mozambique and Tanzania, respectively. However, some studies reported similarly equal prevalence on diarrhoea and non-diarrhoea in DRC (2.3 and 1.7%) and Nigeria (0.5 and 0.0%), respectively (Ogunsanya *et al*., 1994; Wumba *et al*., 2010). These differences can be attributed to variable immune responses of individuals.

### *Giardia* spp. infection in animals

This study has recorded PPE of 17.7% for detection of *Giardia* infections in animals using serological technique which is higher than microscopy and molecular methods. Fayer *et al*. (2012) reported a higher prevalence of 51.1% using molecular technique compared to microscopic methods (19.2%) in the USA. Furthermore, the study by Wang *et al*. (2018) found that both serology (5.8%) and molecular (5.2%) methods had similarly higher detection performance as compared to microscopy (3.7%) for detection of *Giardia* spp. infections in China. On the contrary, Bouzid *et al*. (2015) reported higher *Giardia* spp. infections by microscopy (53.3%) in comparison to molecular (9.2%) and serological methods (26.6%) in Norwich and UK. This difference in diagnostic methods might be due to individual assay sensitivity and less expertise in microscopy.

The subgroup analysis at the country level showed that the PPE of *Giardia* spp. in different African countries ranged from 4.3 to 20.1% with the highest from Nigeria and lowest in Rwanda. These differences might be due to sample size, methodology used to detect and number of studies included in the current study. Animal species were grouped into domestic (cats, cattle, dogs, goats, pigs and sheep) and wildlife (baboons, bonobos, buffaloes, bushbuck, chimpanzee, grasscutter, gorillas, guenons, lions, Maxwell's duiker, monkeys, rabbit, rat, royal antelopes and wild dogs) with each group prevalence pooled together. Our subgroup analysis also revealed that wildlife had lower PPE as compared to domestic animals. However, Castro-Hermida *et al*. (2011) reported wildlife as the main source of exposure for the transmission of *Giardia* parasite to domestic animals, humans, as well as contamination of waterbodies. Our study witnessed a 13.6% increase in the continent PPE of *Giardia* pathogen in animals during the period of 1990–2022. However, periodic analysis revealed a 6.3% initial increase between 1990–2000 and 2001–2010 intervals which was followed by a continuous increase of 7.3% during the 2001–2010 and 2011–2022 intervals. This continuous increase of *Giardia* pathogens might be attributed to failure of animal disease control programmes across the African continent and the use of more advanced diagnostic techniques as years go by.

### *Giardia* spp. infection in waterbodies

Before the year 2000, little interest was shown from researchers within the continent to investigate the prevalence and distribution of *Giardia* spp. in Africa. Interest began to build in the last 2 decades and may be connected to increased resistance of the parasite to chemicals used for water treatment (Jarroll *et al*., 1981; Rice *et al*., 1982; Gerba *et al*., 1993). The prevalence of *Giardia* pathogens measured by molecular methods was higher than microscopic-based methods for most of the environmental waterbodies and varied across countries, with the highest in Tunisia 37.27% and the lowest in Nigeria 15.4%. A study in India has documented equivalent prevalence using molecular (32.0%) and microscopic methods (31.3%) in rivers (Roy *et al*., 2019). However, high prevalence of *Giardia* infection using microscopic method (100.0%) compared to molecular methods (96.2%) in municipal and domestic wastewater in Iran has also been reported (Hatam-Nahavandi *et al*., 2017). This was attributed to the inability of molecular methods to differentiate between viable and non-viable DNA of *Giardia* parasites and lack of expertise in the use of microscope to identify the parasite.

### ‘One Health’ perspective

The ‘One Health’ approach seeks to develop cross-disciplinary relationships to provide more comprehensive responses to diseases that affect multiple species (Lerner and Berg, 2015). A ‘One Health’ concept is becoming more and more necessary, especially in Africa where there is a challenge of access to clean water, and humans living in close contact with animals in rural settlements (Collignon and McEwen, 2019).

This study has reported a consolidated *Giardia* spp. infection prevalence in non-human primates (NHPs), domestic animals, wildlife and waterbodies of the African continent. Furthermore, this study has shown that there is high prevalence of *Giardia* spp. in rural settlements. It is well known that majority of African rural communities practice extensive communal farming which is mainly at livestock–wildlife interface where cattle, goats, pigs and sheep often share pastures and waterbodies with wildlife. In some cases, humans also share the same water sources with animals, hence, the ‘One Health’ concern. Our results highlight the significance of ‘One Health’ concept to comprehend the epidemiology of giardiasis as our study has highlighted the intricate link of the pathogen with human health, the health of both domestic and wild animals, as well as the integrity status of waterbodies. For instance, in China, *G. duodenalis* has been found in large numbers in humans, NHPs, domestic animals, pet animals, wildlife, as well as the environment (Wang *et al*., 2017; Li *et al*., 2023). Only investigations using One Health strategies, which simultaneously consider humans, domestic animals, wildlife and waterbodies, will provide a clear understanding of the key routes of transmission for *Giardia* spp. in Africa. This qualifies giardiasis as one of the diseases in Africa that requires ‘One Health’ approach.

## Conclusion and limitations

Findings of this study suggest that rural population, males, children up to 18 years age, diarrhoeal and HIV+ individuals were subgroups at high risk of getting infected by *Giardia* spp. Whereas for animal demographics, domestic animals were subgroups at high risk of getting infected with *Giardia* spp. We further observed that *Giardia* spp. were also prevalent in NHPs such as chimpanzees, gorillas and monkeys. The robust data presented in this study can be helpful to doctors, veterinarians and environmental scientists by informing them about the epidemiological status of *Giardia* pathogens in humans, animals and waterbodies in Africa.

Our study did not examine the assemblage's diversity of *Giardia* pathogens in humans, animals and waterbodies. Moreover, we only included studies that were published in English language and this language bias has possibly resulted in omission of some relevant studies published in other languages. There were no studies available for *Giardia* parasite infections in Burundi, Cabo Verde, Central African Republic, Congo, Djibouti, Equatorial Guinea, Eswatini, Guinea, Liberia, Mauritania, Mauritius, Niger, Seychelles and South Sudan for humans. In animal studies, some important variables such as sex, stool consistency and age of animals were lacking in included studies. Only 1 study was included in 1980–1990 study interval and there were no studies available in countries such as Angola, Benin, Botswana, Burkina Faso, Burundi, Cabo Verde, Chad, Comoros, Congo, Djibouti, Equatorial Guinea, Eritrea, Eswatini, Gambia, Guinea, Lesotho Liberia, Libya, Madagascar, Malawi, Mali, Mauritania, Mauritius, Morocco, Niger, Sao Tome and Principe, Seychelles, Sierra Leone, Somalia, South Sudan, Sudan, Togo, Tunisia and Zimbabwe.

In studies involving waterbodies, only 1 study was included in the year 1980 until 2000 and no data were available in countries such as Algeria, Angola, Benin, Botswana, Burkina Faso, Burundi, Cabo Verde, Central African Republic, Chad, Comoros, Congo, Djibouti, DR Congo, Equatorial Guinea, Eritrea, Eswatini, Gabon, Gambia, Ghana, Guinea, Guinea-Bissau, Kenya, Lesotho Liberia, Libya, Madagascar, Malawi, Mali, Mauritania, Mauritius, Mozambique, Namibia, Niger, Rwanda, Sao Tome and Principe, Senegal, Seychelles, Sierra Leone, Somalia, South Sudan, Sudan, Togo and Zambia. Finally, we recommend that future studies should be directed to investigation of the epidemiology of this protozoan parasite in countries where surveillance is low as this parasite may pose danger to animals and citizens of those countries.

## Supplementary material

For supplementary material accompanying this paper visit https://doi.org/10.1017/S0031182023000513. click here to view supplementary material
